# Reductions in COQ2 Expression Relate to Reduced ATP Levels in Multiple System Atrophy Brain

**DOI:** 10.3389/fnins.2019.01187

**Published:** 2019-11-01

**Authors:** Jen-Hsiang T. Hsiao, Sivaraman Purushothuman, Poul H. Jensen, Glenda M. Halliday, Woojin Scott Kim

**Affiliations:** ^1^Brain and Mind Centre and Central Clinical School, The University of Sydney, Sydney, NSW, Australia; ^2^School of Medical Sciences, University of New South Wales & Neuroscience Research Australia, Randwick, NSW, Australia; ^3^Department of Biomedicine, DANDRITE-Danish Research Institute of Translational Neuroscience, Aarhus University, Aarhus, Denmark

**Keywords:** multiple system atrophy, parkinsonian disorders, COQ2, COQ7, ATP, coenzyme Q_10_, cerebellum

## Abstract

Multiple system atrophy (MSA) is a progressive neurodegenerative disease clinically characterized by parkinsonism and cerebellar ataxia, and pathologically by oligodendrocyte α-synuclein inclusions. Genetic variants of COQ2 are associated with an increased risk for MSA in certain populations. Also, deficits in the level of coenzyme Q_10_ and its biosynthetic enzymes are associated with MSA. Here, we measured ATP levels and expression of biosynthetic enzymes for coenzyme Q_10_, including COQ2, in multiple regions of MSA and control brains. We found a reduction in ATP levels in disease-affected regions of MSA brain that associated with reduced expression of COQ2 and COQ7, supporting the concept that abnormalities in the biosynthesis of coenzyme Q_10_ play an important role in the pathogenesis of MSA.

## Introduction

Multiple system atrophy (MSA) is a progressive neurodegenerative disease characterized by the clinical triad of parkinsonism, cerebellar ataxia and autonomic dysfunction (Wenning et al., 1997; Ozawa et al., 2001) and pathologically by abnormal α-synuclein deposition in oligodendrocytes, called glial cytoplasmic inclusions (GCIs) (Papp et al., 1989; Spillantini et al., 1997; Gai et al., 1998). The sequence of pathological events in MSA is recognized as oligodendrocyte dysfunction first, followed by neurodegeneration and loss of neurons (Baker et al., 2006; Song et al., 2007; Huang et al., 2008). While the general consensus is that MSA is a highly sporadic disease, emerging evidence has suggested rare genetic variants increase susceptibility, although this appears to be dependent on the geographical distribution of sample patients (Soma et al., 2006; Hara et al., 2007; Stemberger et al., 2011; Multiple-System Atrophy Research Collaboration, 2013).

One such gene is COQ2 (Multiple-System Atrophy Research Collaboration, 2013). COQ2 encodes a biosynthetic enzyme (coenzyme-Q2-polyprenyltransferase) in the production of coenzyme Q_10_, which is required for the production of ATP (Quinzii et al., 2006). Coenzyme Q_10_ is reduced in MSA and not in other parkinsonian disorders, although there is considerable variation in the amount of reduction noted (3–5% versus 40%) (Barca et al., 2016; Schottlaender et al., 2016) and the downstream deficits observed in electron chain transport enzymes (Barca et al., 2016; Schottlaender et al., 2016; Foti et al., 2019). Reductions in coenzyme Q_10_ are likely be due to variations in other biosynthetic enzymes for coenzyme Q_10_ production, as reductions in PDSS1 (the initiating enzyme) and COQ5 have been identified in MSA (Barca et al., 2016; Schottlaender et al., 2016). Interestingly, the level of COQ2 protein was not altered in MSA cerebellum in Tris–HCl buffer (Barca et al., 2016), a tissue fraction unlikely to contain mitochondrial proteins in abundance. To date, whether any of the changes to this pathway in MSA affect ATP production remains unknown. In this study, we assessed whether the levels of ATP are perturbed in MSA and reassessed whether biosynthetic enzymes for coenzyme Q_10_, including COQ2, are involved.

## Materials and Methods

### Human Brain Tissues

Human brain tissues were obtained from the Sydney Brain Bank and NSW Tissue Resource Centre. Ethics approval was from the University of New South Wales Human Research Ethics Committee. Frozen brain tissues from 8 MSA cases and 10 controls were used in this study. MSA brains were clinically and pathologically diagnosed using international diagnostic criteria (Wenning et al., 2004). Controls were free of significant neuropathology. The mean age of MSA cases and controls were 67.7 ± 7.5 and 77.4 ± 8.1 years, respectively. The make up of gender (male/female) was 7/1 and 7/3, respectively. Approximately 50 mg of brain tissue from anatomically specified regions were collected using a 3-mm stainless steel biopsy needle from frozen brain slices (dissected on a bed of dry-ice).

### Detection of GCIs

Formalin-fixed coronal blocks of white matter underlying motor cortex were paraffin-embedded, cut at 10 μm on a microtome, and mounted on 3-aminopropyltriethoxysilane-coated slides. Following pretreatment with 99% formic acid for 3 min, immunoperoxidase staining was performed using antibodies to α-synuclein (mouse mAb42, BD Transduction Labs, United States; diluted 1:100) and an avidin-biotin-peroxidase detection system (Vector Laboratories, Burlingame, CA, United States). Sections were counterstained with 0.5% cresyl violet to identify cell constituents. Labeled sections were evaluated and photographed using an Olympus BX51 fluorescence microscope fitted with specific filter systems and a computerized image analysis system (SPOT camera, Image Pro Plus software).

### ATP Assay

Colorimetric ATP assay was carried out following the manufacturer’s protocol (Abcam, Melbourne, VIC, Australia). Briefly, 10 mg of pulverized frozen tissues were homogenized in 100 μl of ATP assay buffer using a motorized pestle, centrifuged at 13,000 *g* for 5 min at 4°C, and the supernatant collected for analysis. The samples and the standards were added to a 96-well plate containing the ATP Probe reagents and incubated at room temperature in the dark for 30 min. The plate was read using a microplate reader at 570 nm and ATP levels normalized to protein concentration.

### RNA Isolation, Reverse Transcription and Quantitative PCR

RNA was isolated using TRI Reagent (Sigma, Castle Hill, NSW, Australia) following the manufacturer’s protocol from control (*n* = 10) and MSA (*n* = 8) tissues. RNA integrity was assessed with high resolution capillary electrophoresis (Agilent Technologies) and only RNA with RNA Integrity Number value >6.0 was used in the cDNA synthesis. All procedures were carried out using RNase-free reagents and consumables. Five micrograms of RNA was reverse transcribed into cDNA using Moloney-murine leukemia virus reverse transcriptase and random primers (Promega, Annandale, NSW, Australia) in a 20 μl reaction volume. cDNA was used as a template in the quantitative real-time PCR (qPCR) assay, which was carried out using a Mastercycler ep realplex S (Eppendorf) and the fluorescent dye SYBR Green (Bio-Rad), following the manufacturer’s protocol. Briefly, each reaction (20 μl) contained 1× RealMasterMix, 1× SYBR green, 5 pmoles of primers and 1 μl of template. Amplification was carried out with 40 cycles of 94°C for 15 sec and 60°C for 1 min. Gene expression was normalized to the geometric mean of three housekeeper genes, β-actin, GAPDH and glucuronidase-β. The level of expression was calculated using the comparative threshold cycle (Ct) value method using the formula 2^–ΔΔCt^ (where ΔΔCt = ΔCt sample – ΔCt reference).

### Statistical Analysis

MSA and control tissue samples examined were *n* = 8 and *n* = 10, respectively. Data presented are expressed as mean +SE shown by the error bars. Statistical significance was analyzed using the Student’s *t*-test with a *p* < 0.05 considered significant.

## Results and Discussion

### Decreased ATP Levels in MSA Brain

**FIGURE 1:**
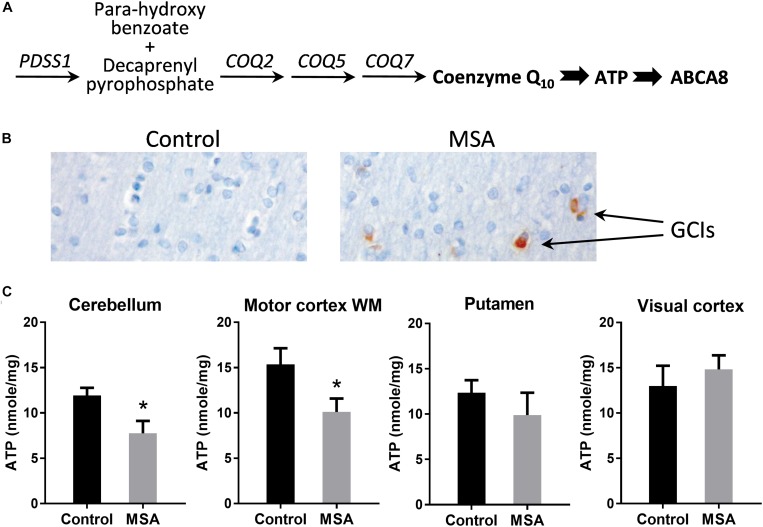
ATP levels in MSA and control brains. **(A)** The biosynthetic pathway of coenzyme Q_10_ and its downstream markers, ATP and ABCA8. **(B)** Immunohistochemistry of cells in the white matter underlying motor cortex using α-synuclein antibody and Nissl staining. **(C)** ATP levels in disease-affected regions (cerebellum, white matter underlying motor cortex and putamen) and disease-unaffected region (visual cortex) of MSA (*n* = 8) and control (*n* = 10) brains. Data represent mean and SE as error bars, ^∗^*p* < 0.05.

Coenzyme Q_10_ is responsible for ATP production (Figure 1A). Here, we analyzed brain tissues from eight clinically and pathologically characterized MSA cases (Wenning et al., 2004) and ten controls without significant neuropathology. Frozen tissue from four specific brain regions were analyzed – disease-affected degenerating gray matter (cerebellum and putamen), disease-affected without significant degeneration (white matter underlying motor cortex) and an unaffected region of the brain (visual cortex). Firstly, we confirmed, by immunohistochemistry, that GCIs were present in the disease-affected regions of MSA brain (Figure 1B). We then measured ATP levels and found that they were significantly decreased in the cerebellum and motor cortex with a non-significant decrease in the putamen, and no differences in the visual cortex (Figure 1C).

*In vivo* magnetic spectroscopic imaging evaluation of ATP levels in the basal ganglia in early MSA patients also showed no significant reduction (Stamelou et al., 2015), but our results suggest that ATP levels are affected in the cerebellar pathways, at least over the disease course. This is consistent with the reliable reduction in coenzyme Q_10_ measured in cerebellar samples, as compared with striatal samples (Barca et al., 2016; Schottlaender et al., 2016). The cerebellum has the highest density of ATP binding sites in the brain (Balcar et al., 1995), using ATP to upregulate synaptic activity (Deitmer et al., 2006). Of note, lower ATP levels are found in the human cerebellum compared with other brain regions (Hetherington et al., 2001) and our data suggests a further reduction in MSA similar to the previously described 30–40% reduction in enzymatic activity in the electron transport chain in MSA (Barca et al., 2016; Schottlaender et al., 2016).

### Decreased COQ2 and COQ7 Expression in MSA Brain

**FIGURE 2:**
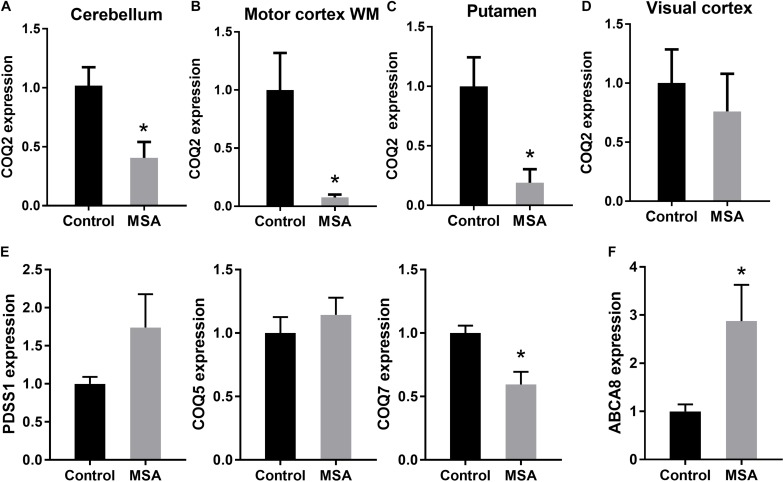
Expression of the main biosynthetic enzymes in coenzyme Q_10_ production. COQ2 mRNA expression in disease-affected regions cerebellum **(A)**, white matter underlying motor cortex **(B)** and putamen **(C)** and disease-unaffected region visual cortex **(D)** of MSA and control brains. **(E)** PDSS1, COQ5 and COQ7 mRNA expression in the cerebellum of MSA and control brains. **(F)** ABCA8 mRNA expression in the cerebellum of MSA and control brains. Data represent mean of MSA (*n* = 8) and control (*n* = 10) brains, and SE as error bars, ^∗^*p* < 0.05.

To account for the decreased ATP levels in MSA, we then analyzed the expression of COQ2 in the same four tissues. COQ2 plays a pivotal role in the biosynthesis of coenzyme Q_10_, combining two essential components, para-hydroxybenzoate and decaprenyl-pyrophosphate (Figure 1A). We found that COQ2 mRNA levels were significantly decreased in MSA compared to controls in all three disease-affected regions and unaltered in the visual cortex (Figure 2A). In addition, we analyzed the expression of PDSS1, COQ5, and COQ7 mRNAs in the cerebellum. We found that COQ7 mRNA levels were significantly decreased in MSA compared to controls, whereas PDSS1 and COQ5 mRNA levels were unchanged (Figure 2B). While this contrasts with previous data using the most soluble protein fraction of MSA cerebellar tissue (Barca et al., 2016; Schottlaender et al., 2016), our data show significantly reduced expression of COQ2 and COQ7 mRNAs and not PDSS1 and COQ5 mRNAs in this region in MSA. The loss of soluble PDSS1 and COQ5 protein described previously (Barca et al., 2016; Schottlaender et al., 2016) may reflect a compensatory redistribution of these proteins to mitochondrial tissue fractions. Importantly, we found that COQ2 (Spearman’s correlation = 0.489; *P* < 0.05) and COQ7 (Spearman’s correlation = 0.555; *P* < 0.05) expression levels were correlated to ATP levels in the cerebellum, supporting a relationship between reductions in COQ2 and COQ7 mRNA levels and ATP levels in MSA tissue. Furthermore, COQ2 and COQ7 expression levels were correlated to each other (Spearman’s correlation = 0.535; *P* < 0.05).

To further investigate the impact of decreased COQ2/COQ7 mRNA levels on ATP production in MSA, we measured the expression of the ATP-dependent gene ABCA8 (ATP Binding Cassette A8), which is implicated in oligodendrocyte lipid dysregulation in MSA pathogenesis (Bleasel et al., 2013; Kim et al., 2013). We found that ABCA8 mRNA expression was significantly upregulated in MSA cerebellum compared to controls (Figure 2C) as a feedback response to the decreased ATP levels in MSA brain. When put together, these data suggest that the biosynthetic enzymes involved in the coenzyme Q_10_ production are specifically altered in disease-affected regions of MSA brain, resulting in the reduction of coenzyme Q_10_ and ATP levels, and a subsequent alteration in ATP-dependent activity. These results support further investigation into the role of biosynthetic enzymes in coenzyme Q_10_ production in MSA pathogenesis.

## Data Availability Statement

The datasets generated for this study are available on request to the corresponding author.

## Ethics Statement

The studies involving human participants were reviewed and approved by the University of New South Wales Human Research Ethics Committee. The patients/participants provided their written informed consent to participate in this study.

## Author Contributions

WK conceived and designed the study. WK and GH wrote the manuscript. All authors participated in the acquisition and analysis of the data.

## Conflict of Interest

The authors declare that the research was conducted in the absence of any commercial or financial relationships that could be construed as a potential conflict of interest.

## References

[B1] Baker K. G. Huang Y. Mccann H. Gai W. P. Jensen P. H. Halliday G. M. (2006). P25alpha immunoreactive but alpha-synuclein immunonegative neuronal inclusions in multiple system atrophy. *Acta Neuropathol.* 111 193–195. 10.1007/s00401-005-0008-x 16421739

[B2] Balcar V. J. Li Y. Killinger S. Bennett M. R. (1995). Autoradiography of P2x ATP receptors in the rat brain. *Br. J. Pharmacol.* 115 302–306. 10.1111/j.1476-5381.1995.tb15877.x 7670731 PMC1908314

[B3] Barca E. Kleiner G. Tang G. Ziosi M. Tadesse S. Masliah E. (2016). Decreased coenzyme Q10 levels in multiple system atrophy cerebellum. *J. Neuropathol. Exp. Neurol.* 75 663–672. 10.1093/jnen/nlw037 27235405 PMC4913434

[B4] Bleasel J. M. Hsiao J. H. Halliday G. M. Kim W. S. (2013). Increased expression of ABCA8 in multiple system atrophy brain is associated with changes in pathogenic proteins. *J. Parkins. Dis.* 3 331–339. 10.3233/JPD-130203 23948991

[B5] Deitmer J. W. Brockhaus J. Casel D. (2006). Modulation of synaptic activity in purkinje neurons by ATP. *Cerebellum* 5 49–54. 10.1080/14734220500497456 16527764

[B6] Foti S. C. Hargreaves I. Carrington S. Kiely A. P. Houlden H. Holton J. L. (2019). Cerebral mitochondrial electron transport chain dysfunction in multiple system atrophy and Parkinson’s disease. *Sci. Rep.* 9:6559. 10.1038/s41598-019-42902-7 PMC6484105 31024027

[B7] Gai W. P. Power J. H. Blumbergs P. C. Blessing W. W. (1998). Multiple-system atrophy: a new alpha-synuclein disease? *Lancet* 352 547–548. 10.1016/s0140-6736(05)79256-4 9716068

[B8] Hara K. Momose Y. Tokiguchi S. Shimohata M. Terajima K. Onodera O. (2007). Multiplex families with multiple system atrophy. *Arch. Neurol.* 64 545–551. 17420317 10.1001/archneur.64.4.545

[B9] Hetherington H. P. Spencer D. D. Vaughan J. T. Pan J. W. (2001). Quantitative (31)P spectroscopic imaging of human brain at 4 Tesla: assessment of gray and white matter differences of phosphocreatine and ATP. *Magn. Reson. Med.* 45 46–52. 10.1002/1522-2594(200101)45:1<46::aid-mrm1008>3.0.co;2-n 11146485

[B10] Huang Y. Song Y. J. Murphy K. Holton J. L. Lashley T. Revesz T. (2008). LRRK2 and parkin immunoreactivity in multiple system atrophy inclusions. *Acta Neuropathol.* 116 639–646. 10.1007/s00401-008-0446-3 18936941

[B11] Kim W. S. Hsiao J. H. Bhatia S. Glaros E. N. Don A. S. Tsuruoka S. (2013). ABCA8 stimulates sphingomyelin production in oligodendrocytes. *Biochem. J.* 452 401–410. 10.1042/BJ20121764 23560799

[B12] Multiple-System Atrophy Research Collaboration (2013). Mutations in COQ2 in familial and sporadic multiple-system atrophy. *N. Engl. J. Med.* 369 233–244. 10.1056/NEJMoa1212115 23758206

[B13] Ozawa T. Okuizumi K. Ikeuchi T. Wakabayashi K. Takahashi H. Tsuji S. (2001). Analysis of the expression level of alpha-synuclein mRNA using postmortem brain samples from pathologically confirmed cases of multiple system atrophy. *Acta Neuropathol.* 102 188–190. 11563635 10.1007/s004010100367

[B14] Papp M. I. Kahn J. E. Lantos P. L. (1989). Glial cytoplasmic inclusions in the CNS of patients with multiple system atrophy (striatonigral degeneration, olivopontocerebellar atrophy and Shy-Drager syndrome). *J. Neurol. Sci.* 94 79–100. 10.1016/0022-510x(89)90219-0 2559165

[B15] Quinzii C. Naini A. Salviati L. Trevisson E. Navas P. Dimauro S. (2006). A mutation in para-hydroxybenzoate-polyprenyl transferase (COQ2) causes primary coenzyme Q10 deficiency. *Am. J. Hum. Genet.* 78 345–349. 10.1086/500092 16400613 PMC1380241

[B16] Schottlaender L. V. Bettencourt C. Kiely A. P. Chalasani A. Neergheen V. Holton J. L. (2016). Coenzyme Q10 levels are decreased in the cerebellum of multiple-system atrophy patients. *PLoS One* 11:e0149557. 10.1371/journal.pone.0149557 26894433 PMC4760984

[B17] Soma H. Yabe I. Takei A. Fujiki N. Yanagihara T. Sasaki H. (2006). Heredity in multiple system atrophy. *J. Neurol. Sci.* 240 107–110. 10.1016/j.jns.2005.09.003 16307759

[B18] Song Y. J. Lundvig D. M. Huang Y. Gai W. P. Blumbergs P. C. Hojrup P. (2007). p25alpha relocalizes in oligodendroglia from myelin to cytoplasmic inclusions in multiple system atrophy. *Am. J. Pathol.* 171 1291–1303. 10.2353/ajpath.2007.070201 17823288 PMC1988878

[B19] Spillantini M. G. Schmidt M. L. Lee V. M. Trojanowski J. Q. Jakes R. Goedert M. (1997). Alpha-synuclein in Lewy bodies. *Nature* 388 839–840. 9278044 10.1038/42166

[B20] Stamelou M. Pilatus U. Reuss A. Respondek G. Knake S. Oertel W. H. (2015). Brain energy metabolism in early MSA-P: a phosphorus and proton magnetic resonance spectroscopy study. *Parkins. Relat. Disord.* 21 533–535. 10.1016/j.parkreldis.2015.03.001 25801909

[B21] Stemberger S. Scholz S. W. Singleton A. B. Wenning G. K. (2011). Genetic players in multiple system atrophy: unfolding the nature of the beast. *Neurobiol. Aging* 32:1924.e5-e14. 10.1016/j.neurobiolaging.2011.04.001 21601954 PMC3157605

[B22] Wenning G. Tison F. Ben Shlomo Y. Daniel S. Quinn N. (1997). Multiple system atrophy: a review of 203 pathologically proven cases. *Mov. Disord.* 12 133–147. 10.1002/mds.870120203 9087971

[B23] Wenning G. K. Tison F. Seppi K. Sampaio C. Diem A. Yekhlef F. (2004). Development and validation of the unified multiple system atrophy rating scale (UMSARS). *Mov. Disord.* 19 1391–1402. 10.1002/mds.20255 15452868

